# Case Report: The Emerging Role of Ring Chromosome 22 in Phelan-McDermid Syndrome With Atypical Teratoid/Rhabdoid Tumor: The First Child Treated With Growth Hormone

**DOI:** 10.3389/fneur.2021.741062

**Published:** 2021-10-29

**Authors:** Marco Crocco, Marta Panciroli, Claudia Milanaccio, Cristina Morerio, Antonio Verrico, Maria Luisa Garrè, Natascia Di Iorgi, Valeria Capra

**Affiliations:** ^1^Neuro-Oncology Unit, Istituto di Ricovero e Cura a Carattere Scientifico Giannina Gaslini Institute, Genoa, Italy; ^2^Department of Neuroscience, Rehabilitation, Ophthalmology, Genetics, Child and Maternal Health, University of Genova, Genoa, Italy; ^3^Laboratory of Human Genetics, Istituto di Ricovero e Cura a Carattere Scientifico Giannina Gaslini Institute, Genoa, Italy; ^4^Department of Pediatrics, Istituto di Ricovero e Cura a Carattere Scientifico Giannina Gaslini Institute, Genoa, Italy; ^5^Medical Genetics Unit, Istituto di Ricovero e Cura a Carattere Scientifico Giannina Gaslini Institute, Genoa, Italy

**Keywords:** atypical teratoid/rhabdoid tumor, growth hormone, *SMARCB1*, *INI1*, ring chromosome, midazolam, case report, Phelan McDermid Syndrome (PHMDS)

## Abstract

Atypical teratoid/rhabdoid tumors (AT/RTs) in the rhabdoid tumor predisposition syndromes are most often caused by germline mutations of the *SMARCB1* gene located in chromosome 22q11.2. Although rarely, it can also result from the constitutional ring chromosome 22 (r22): during mitosis the ring chromosome may lead to an increased rate of somatic mutations, resulting in rhabdoid tumor predispositions when the tumor-suppressor gene *SMARCB1* is involved. Individuals with r22 may present similar features as those with Phelan-McDermid syndrome (PMDS) due to 22q13.3 deletion, including the *SHANK3* gene. Despite several reports on AT/RT in children with r22 and/or PMDS have been published, the role of constitutional r22 as new oncogenic mechanism for AT/RT is still under investigation. There is not a lot of data available on therapeutic and prognostic implications of r22 in AT/RT and PMDS. Herein, we present the first case of a child with constitutional r22, PMDS and AT/RT of the brain, who is a long term survivor and is been treated with growth hormone. We also describe an unexpected adverse reaction to midazolam.

## Introduction

Atypical teratoid/rhabdoid tumors (AT/RTs) represent 20% of highly malignant tumors of the central nervous system (CNS), in children <3 years old (1).

The *SMARCB1/INI1* gene codes a subunit of the switch/sucrose non-fermentable (SWI/SNF) complex, actin-dependent chromatin remodeling complex and is known to act as a tumor suppressor. This gene is frequently inactivated through somatic point mutation or deletion, followed by a second hit. The constitutional pathogenic variant in *SMARCB1* causes rhabdoid tumor predisposition syndrome. About 25%–35% of newly diagnosed children with rhabdoid tumors have a germline pathogenic variant in *SMARCB1* (2). The rhabdoid tumor predisposition syndrome is rarely as a consequence of the loss of the expression of the ATPase subunit SMARCA4, also known as BRG1, another SWI/SNF chromatin-remodeling complex member (3). Therefore, the presence of the expression of SWI/SNF-related matrix-associated actin-dependent regulator of chromatin subfamily B member 1 (SMARCB1) protein does not rule out a diagnosis of AT/RT (4).

AT/RT has been previously reported in patients with r22 (5–8) and recently also in PMDS (OMIM# 606232) (9–11). Ring chromosome 22 syndrome is an autosomal anomaly with most features common to 22q13 deletion syndrome. Byers et al. (11) hypothesized that mitotic instability of r22 could lead to somatic mosaic aneuploidy, resulting in a heterozygous loss of *SMARCB1*. A complete loss of *SMARCB1* expression in tumor cells can be the result of a second-hit or an alternative inactivating mechanism, such as methylation pattern, variants in non-coding regions, promoters or enhancers.

PMDS is a contiguous gene disorder caused by a heterozygous contiguous gene deletion at chromosome 22q13 or by mutation in the *SHANK3* gene, which is located within the minimum critical region. The loss of 22q13.3 can result from a simple deletion, translocation, ring chromosome formation or, less commonly, from structural changes affecting the long arm of chromosome 22, specifically the region containing the *SHANK3* gene. It is underdiagnosed and till now the prevalence is unknown (12). More than 50% of patients show autism or autistic-like behavior due to haploinsufficiency of the synaptic scaffolding protein SHANK3. Other major features of PMDS include: global developmental delay, neurologic and motor regression with moderate to severe intellectual impairment, absent or severely delayed speech, hypotonia, dysmorphic features, normal to accelerated growth and increased pain tolerance (13).

In cellular models of PMDS, neurons with reduced *SHANK3* expression show defects in excitatory, but not inhibitory, synaptic transmission. Excitatory synaptic transmission can be corrected by restoring *SHANK3* expression or by treating neurons with insulin-like growth factor 1 (IGF-1) leading to the formation of mature excitatory synapses that lack SHANK3 but contain the post synaptic density protein-95 (PSD-95) and N-methyl-D-aspartate (NMDA) receptors.

In the literature, patients with autism spectrum disorders or PMDS do not show an increased incidence of adverse events post-procedural sedation with midazolam (14, 15). Li et al. have examined whether sensitivity to isoflurane anesthesia is altered in genetic mouse model of PMDS (*Shank3* deleted), they showed that *Shank3* deletion confers enhanced sensitivity to isoflurane (16).

Herein, we present from a multidisciplinary perspective a complex case of r22 in a child with PMDS due to a partial deletion of chromosome 22 not involving the *SMARCB1* gene, who survived a metastatic AT/RT of the CNS. We also describe, for the first time in such a patient, the benefits of hrGH therapy and a serious adverse event after procedural sedation with midazolam.

## Case Presentation

**Figure 1:**
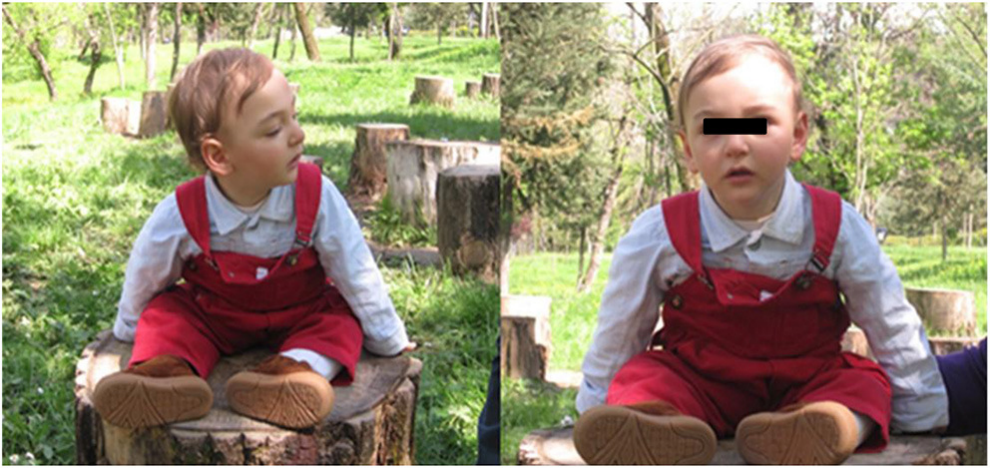
One year old.

**Figure 2:**
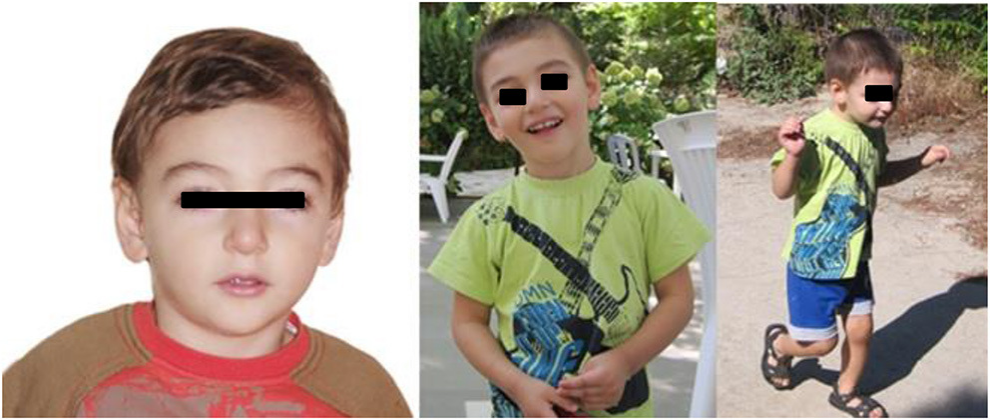
Two years old.

The child was born after a 38-week uncomplicated pregnancy from non-consanguineous parents. The family history was uneventful. APGAR (Appearance, Pulse, Grimace, Activity, Respiration) scores were 9 at the 1st min and 10 at 5th min. After delivery, the neonate did not present major dysmorphisms or hypotonia; normal growth and development were reported (Figures 1, 2).

At 2 years and 6 months the child started having feeding problems and vomiting episodes and he underwent a brain Magnetic Resonance Imaging (MRI) that showed diffused pathological tissue of the 4th ventricle area with metastatic diffusion at the left temporal brain lobe, spinal cord at D5-D6 level and cauda.

**Figure 3:**
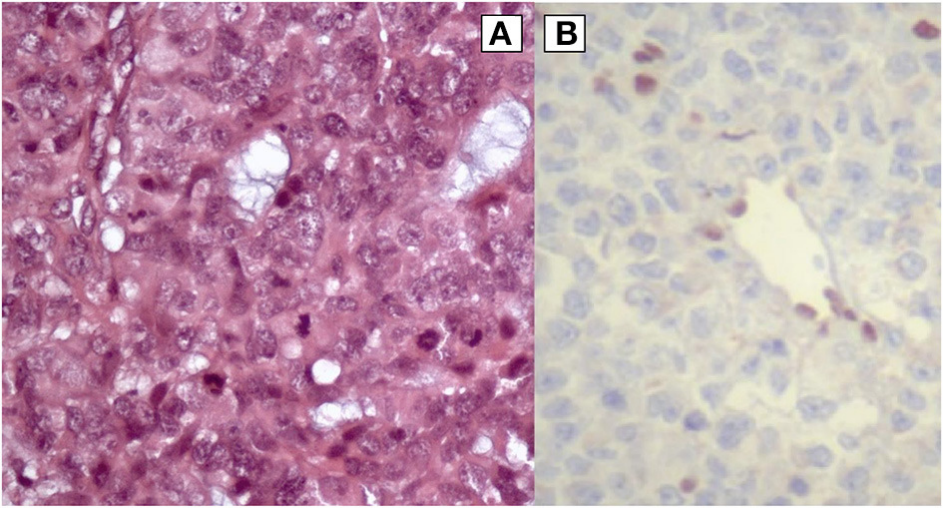
**(A)** High-power view illustrates AT/RT cells of the patient (hematoxylin and eosin staining). **(B)** Rhabdoid cells negative for SMARCB1 protein (immunohistochemistry).

Partial resection of the mass occupying the 4th ventricle allowed the diagnosis of AT/RT. Histological analysis of the tumor showed a population of cells with rhabdoid features (Figure 3A). Immunohistochemistry showed the absence of the expression of SMARCB1 protein (Figure 3B), at the same time the expression of EMA and focal of AML and SYN was observed (data not shown). The histological diagnosis was confirmed by a national centralized reviewer. An abdominal ultrasound ruled out the presence of any renal mass.

After neurosurgery, the patient's clinical conditions were very severe due to the progression of the spinal metastasis, with pain difficult to control, progressive paraparesis, sphincter impairment and posterior fossa syndrome. Chemotherapy (CT) was started according to European protocol EU RHAB 2010 and adapted for the critical clinical conditions. Three courses of induction CT (Doxorubicin 1.4 mg/kg/24 h × 2 days; Vinorelbine 0.7 mg/kg for 3 days; 2 ICE cycles: Ifosfamide 66.6 mg/kg and Etoposide 3.3 mg/kg x 3 days, Carboplatinum 16.7 mg/kg for 1 day) and 2 courses of myeloablative high dose CT (1 course of Thiotepa 300 mg/m^2^ x 3 days + Carbo 500 mg/m^2^ x 3 days and 1 course of Thiotepa 300 mg/m^2^ x 3 days) were delivered. The MRI showed that all the sites exhibited a good response after the induction-and myeloablative-CT. Treatment was completed at the age of 3 years and 2 months when the child had been irradiated by Tomotherapy technique: 3520 cGy on the cranio-spinal axis, followed by boosts on sites of residual disease: posterior fossa and temporo-polar lesions up to 5800 cGy; cauda equina up to 4960 cGy; D5-D6 4960 up to cGy.

After irradiation, the child suffered prolonged leukopenia and thrombocytopenia with one episode of general infection treated with multiple transfusions and broad-spectrum antibiotics. Valproic Acid was chosen as a prophylactic anti-seizure drug for its anti-tumor properties in high-grade brain tumors (17, 18).

The MRI at 1 and 3 months after irradiation showed a complete remission at the level of the primary site, while millimetric cystic lesions improved slightly (with signal like cerebrospinal fluid) at the temporo-polar site and at the residual spinal lesions. The MRI, 6 months after the therapies, showed a definite improvement of the metastatic spinal lesions, which were no longer detectable.

Despite starting the intensive multidisciplinary rehabilitation program early, the patient exhibited the following signs: growth and weight impairment; severe psychomotor regression; global hypotonia; cognitive impairment with only partial recovery after rehabilitation; oropharyngeal dysphagia for liquid and solid food; osteoporosis and low trauma vertebral fractures; and alopecia.

Three years after the end of radiotherapy, the child had a complete neuroradiologic remission of the disease, and the patient met auxological criteria to initiate evaluation for growth hormone deficiency (GHD). Even though the height was appropriate for age, sex and target height, it dropped from +0.8 to−0.3 SDS (Standard Deviation Score) due to the pathological growth velocity (<2 cm in 1 year). Two provocative GH tests confirmed the diagnosis of GHD (the peak value of serum GH was 3,52 ng/mL after stimulation with intravenous infusion of Arginine, and 6.63 ng/mL after stimulation with intramuscular injection of Glucagone; in both tests the normal values were >8 ng/ml). A full evaluation of the other pituitary hormones ruled out further damages. Recombinant human GH (hrGH) was started at a 0.028 mg/kg/day dose without adverse effects. After 6 months of hrGH, the 1 year growth velocity increased from 4 cm/year (−2.2 SDS) to 5.5 cm/year (−0.3 SDS) and the parents reported an improved quality of life, muscle strength and physical capability. At the last evaluation, after 18 months of treatment, the growth velocity remained consistently regular (6.2 cm/year, −0.8 SDS); IGF-1 increased from 47.6 ng/ml (−3.8 SDS) to 78.1 ng/ml (−2.3 SDS) 6 months after hrGH initiation (0.028 mg/kg/day), reaching normal values up to 146.2 ng/ml (−0.55 SDS) after a further 18 months of treatment (0.031 mg/kg/day). The latter dose is currently ongoing without side effects.

As for institutional protocol, intranasal midazolam was implemented to increase the patient's compliance to procedural sedation for MRI; shortly after midazolam was administered for the first time to our patient (months before starting hrGH) he showed signs of shock (severe hypotension, prolonged capillary refill time, a decreased level of consciousness and urinary output, and bradycardia) and needed resuscitation. The hypovolemic shock treatment included three intravenous boluses of fluids 20 ml/kg (5% dextrose in NaCl 0.9% physiological solution), and a hydrocortisone 10 mg/kg intravenous injection which resulted in a slow recovery of the state of consciousness and improvement of the vital signs. A subsequent ACTH (adrenocorticotropic hormone) low dose test excluded central adrenal insufficiency. An MRI was performed 5 months after starting hrGH using Propofol intravenously as procedural sedation without side effects. Complete remission of the disease was confirmed after 18 months of hrGH therapy (5 years after the end of oncological treatment).

**Figure 4:**
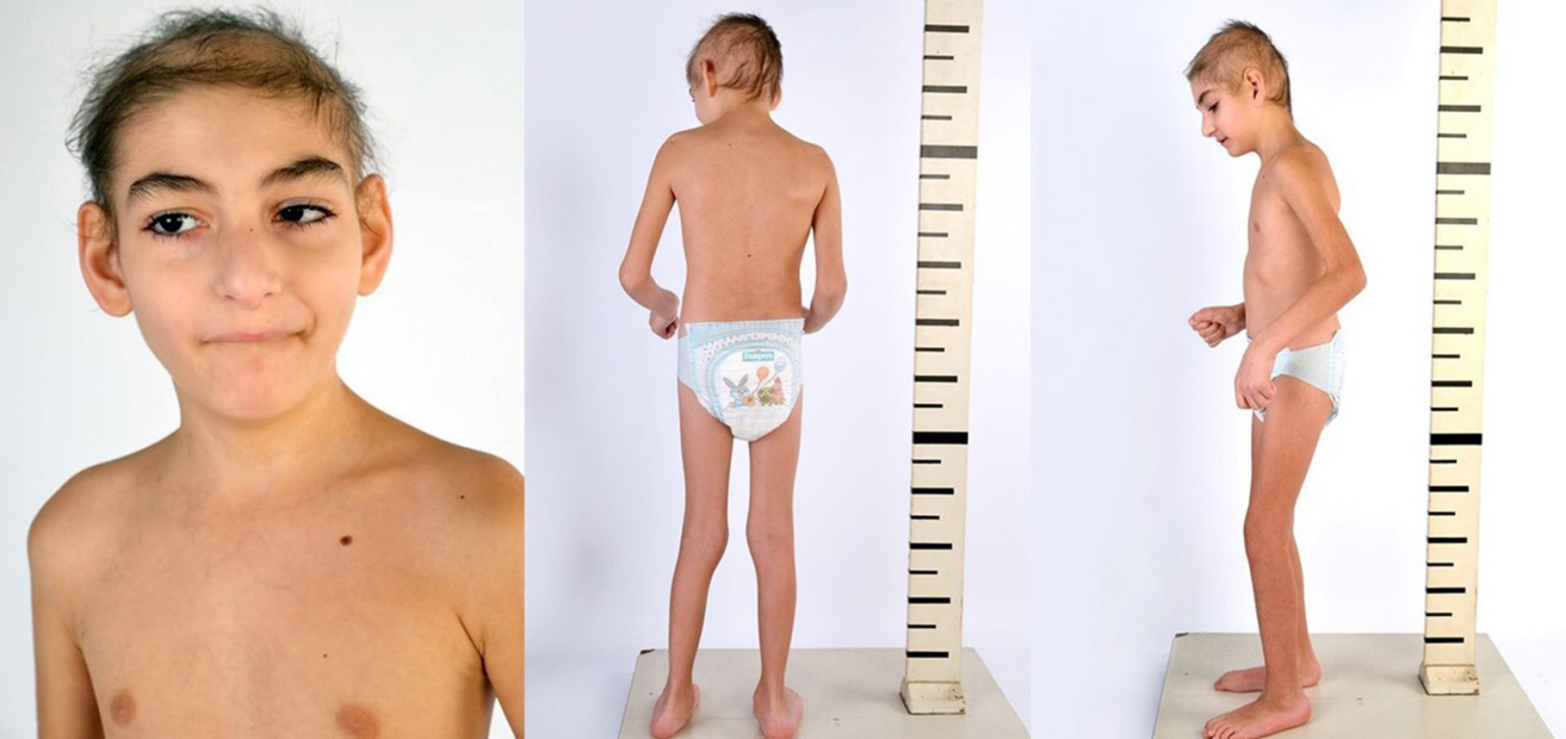
Eight years old.

**Figure 5:**
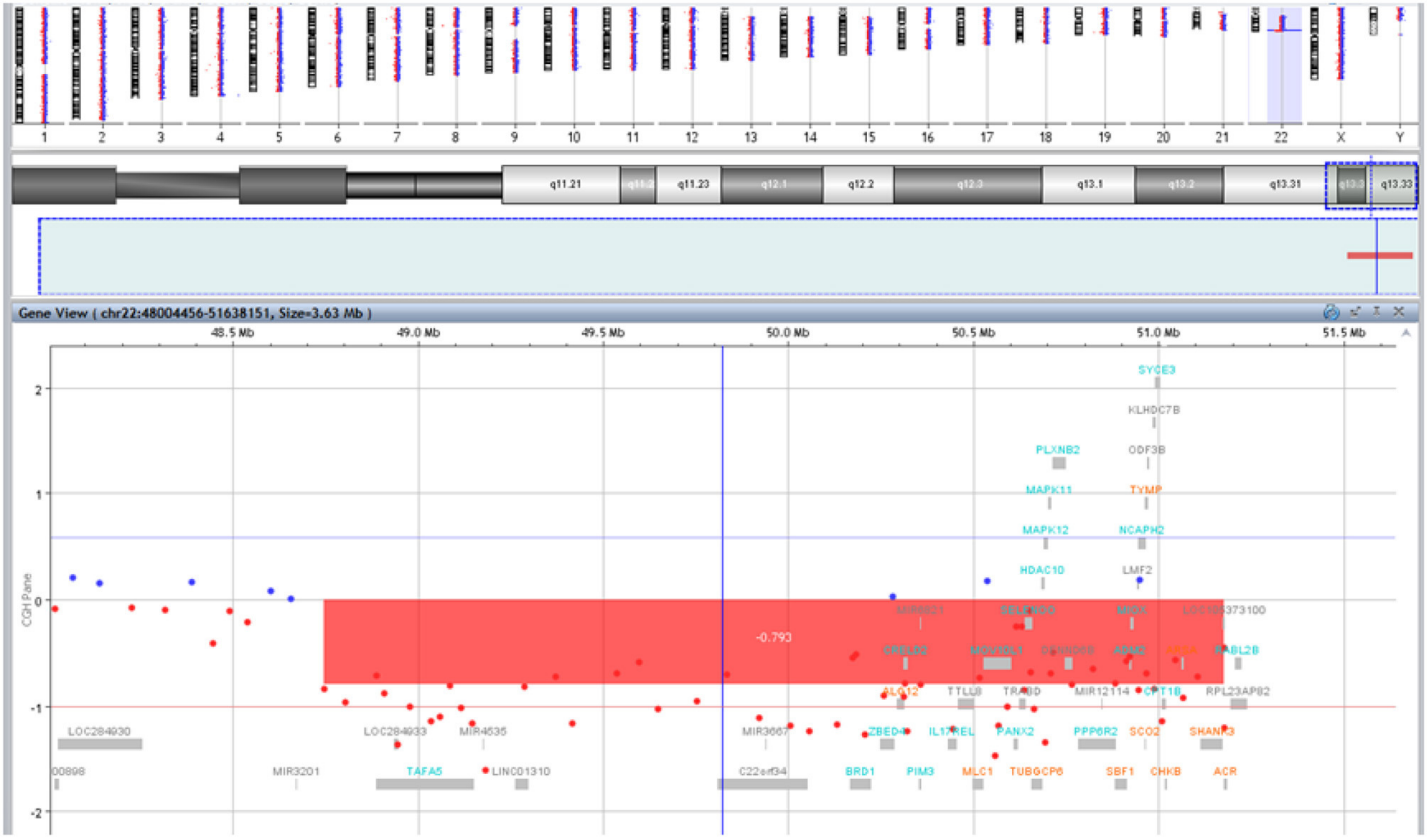
Results of array-CGH analysis. Zoom view of long arm of chromosome 22 shows a 2,4 Mb deletion at 22q13.32q13.33 spanning from position 48746241 bp to the telomere.

**Figure 6:**
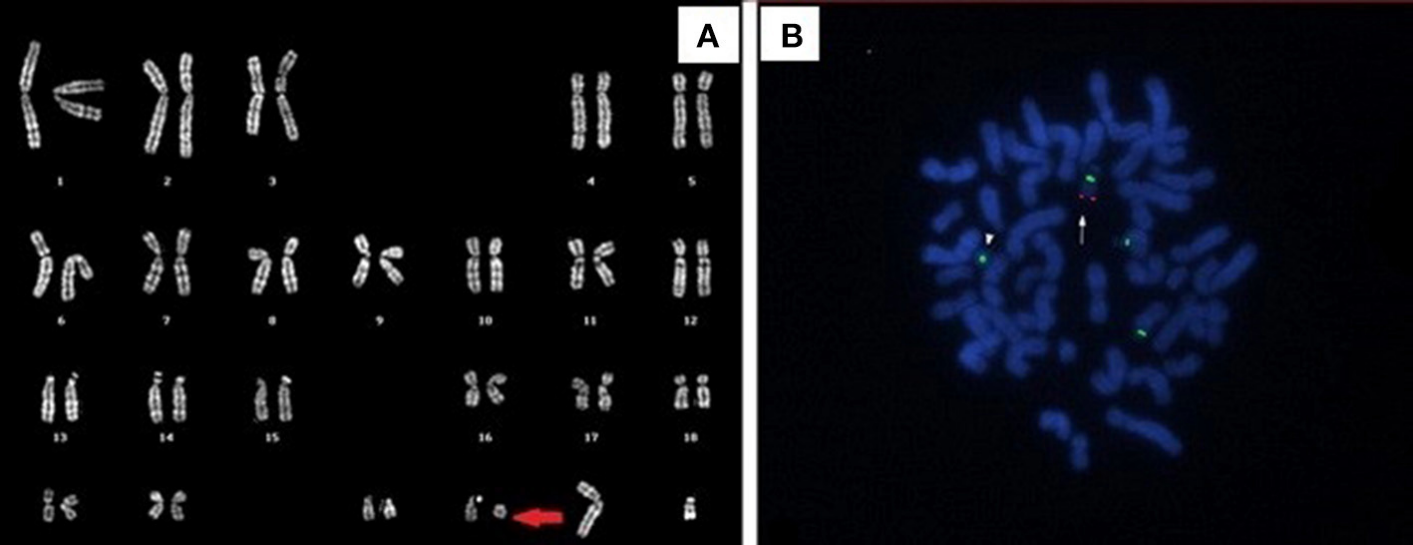
**(A)** Q-banded karyotype showing ring chromosome 22. **(B)** FISH analysis with 14/22 centromeric probe and specific *SHANK3* probe (green and red signals, respectively). The arrow indicates the normal chromosome 22, the arrowhead shows the absence of *SHANK3* signal on the ring chromosome 22.

Mild dysmorphic facial features were detectable by facial photos before chemo-radiotherapy (long eyelashes, bulbous nose, large and prominent ears) (Figure 2) and due to the severe late effects (Figure 4) the child underwent genetic analysis. Array-CGH (Whole-genome 60 K Agilent array, Human Genome CGH Microarray, Agilent Technologies, Santa Clara, CA, USA) revealed a *de-novo* chromosomal rearrangement: arr[GRCh37] 22q13.32q13.33(48746241_tel)x1 dn involving a partial deletion of long arm of chromosome 22 that is extended for 2,4 Mb from nucleotide 48.746.241 to the telomere (Figure 5). Data was analyzed using Agilent Cytogenomics Software. All genomic positions were reported according to the human genome assembly (GRCh37/hg19). This genomic region is known to include 36 genes (including *SHANK3*) and this chromosomic region is responsible for the deletion syndrome of 22q13.3. The presence of the *SHANK3* deletion confirmed the diagnosis of PMDS. *SMARCB1* was not mutated in the germline DNA as demonstrated by the Next-Generation Sequencing analysis (Ion Torrent NGS sequencing systems, Ion AmpliSeq kit, Waltham, MA, USA), the MLPA analysis was not possible due to the lack of biological material available. Cytogenetic analysis using Q-banding techniques at the 400 bands of resolution revealed a 46, XY, r22(p11.2;q13.32)dn karyotype in the 30 metaphase cells examined (Figure 6A). FISH studies were performed using SureFISH probe Agilent (Santa Clara, CA, USA) 22q13.33 SHANK3 and chromosome 14/22 alpha satellite probe Acquarius Cytocell (Cambridge, UK) (Figure 6B), the results confirmed the CGH Microarray and karyotype analysis. The genetic study of both parents was negative.

## Discussion

In our clinical case *SMARCB1* was not mutated in the germline DNA and the partial deletion of chromosome 22 not involving the *SMARCB1* gene, nevertheless the immunohistochemistry of the AT/RT tumor of our patient demonstrated the absence of the SMARCB1 protein (Figure 3B), suggesting the loss of both alleles. We hypothesized that in cancer cells the loss of expression of the SMARCB1 protein was due to a somatic mosaic aneuploidy involving r22 and including *SMARCB1*, as described by Byers et al. (11), followed by a second-hit.

Familial cases described as the rhabdoid tumor predisposition syndrome, have been linked to heterozygous *SMARCB1* germline mutations. The loss of expression of *SMARCB1* is the defining molecular genetic feature of AT/RT (19, 20). Immunohistochemical staining of SMARCB1 is currently considered very sensitive and highly specific for the detection of *SMARCB1* genetic defects (21). Tsai et al. (22), by using gene expression microarray analysis on Taiwanese AT/RT, found that four of the five AT/RT cases examined still showed positive SMARCB1 mRNA signal even though SMARCB1 proteins were stained negative by immunohistochemistry. They sequenced the whole *SMARCB1* gene in 4 AT/RT cases (no DNA available for the fifth case) to check DNA mutations in the *SMARCB1* gene genomic DNA region. Three of the four AT/RTs did not show sequence alternation in the *SMARCB1* gene after the PCR-amplified genomic DNAs were isolated from fresh frozen tumor tissue. Similarly, in another independent cohort containing 20 AT/RTs, about 10% of cases did not have coding sequence mutations in any of the 9 exons of *SMARCB1* and yet had decreased expression levels of SMARCB1 by RT-PCR analysis and undetectable levels of the protein by Western blot analysis (23).

The monosomy of 22q13.3 has findings typical of such deletion syndrome characterized by global developmental delay, generalized hypotonia, and absent or severely delayed speech (24), but the true prevalence of 22q13.3 deletion may be underestimated (12). The deleted region contains numerous genes that could have an effect on the presence of AT/RT and the developmental delay of the child.

Among the genes, the bromodomain containing 1 (*BRD1*) gene has been shown to be associated with schizophrenia and bipolar disorder in genetic studies, including gene-wise significant association in a large schizophrenia genome-wide association study meta-analysis (25, 26). Furthermore, Cai QQ et al. demonstrated that sulfatide interaction with the *BRD1* mediates acetylation and is important for the regulation of integrin αV gene expression in hepatocarcinoma (27) and similarly could have the same effect in AT/RT. While proviral integration of Moloney murine 3 (*PIM3*) is associated with Distal Muscular Dystrophy, it has also been demonstrated to function as an oncogenic factor promoting tumor growth in colorectal cancer (28) and hepatoblastoma (29). The modulator of VRAC current 1 (*MLC1*) is associated with Megalencephalic Leukoencephalopathy with subcortical cysts and it could be related to melanoma survival and chemoresistance through mitogen-activated protein kinases (*MAPK*) - extracellular signal-regulated kinases (*ERK*) pathway (30, 31). The histone deacetylase 10 (*HDAC10*) is involved in transcriptional repression of the histone acetyl transferase (*HAT*). In many cancers, the balance between *HAT* and *HDAC* is altered. Diseases associated with *HDAC10* include Neuroblastoma (32), renal cell carcinoma (33) and lung adenocarcinoma (34). HDAC inhibitors (HDACis) were shown to exert antitumor effects in several cancer cell lines, confirming the role of HDACs in oncogenesis (35). *MAPK8IP2* gene encodes a scaffold protein: The JNK-interacting protein (JIP), that is more abundantly expressed in the cerebellum, pituitary gland, occipital lobes and the amygdala, and it could be associated to Ameloblastic Carcinoma and Spinocerebellar Ataxia X-Linked 5. The p38-*MAPK* signaling, including p38β (*MAPK11*) and p38γ (*MAPK12*), is associated with the development and progression of several types of cancer (36–38). On the other hand, the MAPK pathway could be involved in the occurrence of AT/RT (39, 40). *PLXNB2* is required for normal differentiation and migration of neuronal cells during brain corticogenesis and for normal embryonic brain development (41–43). Therefore, also PLXNB2 could contribute to the delay in developmental of the child.

*SHANK3* is a protein coding gene and a member of the *SHANK* gene family. The SHANK3 protein is particularly involved in the postsynaptic density that connects neurotransmitter receptors, ion channels, and other membrane proteins to the actin cytoskeleton and G-protein-coupled signaling pathways. Mutations in the *SHANK3* gene are a cause of autism spectrum disorder, which is characterized by impairments in social interaction and communication, and restricted behavioral patterns and interests. Mutations in this gene are also associated with schizophrenia type 15, and are a major cause in the neurological symptoms of PMDS (9), that could be related to the severe clinical neurological features that characterize the child. While *SHANK3* has been extensively studied as the cause (or at least the main contributory gene) of PMDS, many other genes are involved in the pathogenesis of this contiguous gene disorder and its phenotype. Despite the fact that *SHANK3* is thought to be the strongest candidate gene for the neurological features of PMDS (24, 44, 45), interstitial deletions of the 22q13 chromosomal region (that do not involve *SHANK3*) have rarely been detected in patients with the main clinical features common to PMDS (46, 47). These patients display a phenotype partially overlapping with PMDS, including developmental delay, hypotonia and language disorders, but the autism spectrum disorder symptomatology was reported less frequently in these patients (48).

Neurological impairment is a common late effect in central nervous system tumors; in contrast, it's early detection in our patient prompted genetic counseling. The diagnosis of the PMDS, a contiguous gene disorder, could explain the rapidly worsening neurological effects after cancer therapies, and the failure of multidisciplinary rehabilitation, despite the favorable trend of the oncological disease. It is possible that ring chromosome has important prognostic implications playing a role in worsening the effects of the cancer therapies, especially radiotherapy, due to the continuous risk of dynamic somatic mutations (11, 49, 50).

Late effects due to hypothalamic-pituitary irradiation usually begins with GH deficiency. The endocrine-metabolic effects of hrGH and insulin-like growth factor-1 (IGF-1) therapy are well known in patients with pituitary defects and primary IGF-1 deficiency, respectively. In recent years, neurologic and other effects in Phelan McDermid Syndrome have been studied after IGF-1, but not after hrGH therapy. Recent evidence in mice and human neuronal models of *SHANK3* deficiency (51, 52) suggest that IGF-1 can reverse synaptic plasticity and motor learning deficits. This evidence was also supported by Kolevzon et al. (53) in a placebo-controlled, double-blind, crossover study in which nine children with PMDS aged 5 to 15 years were treated with IGF-1or placebo for 3 months in a random order and crossed to the alternative treatment arm after a 4 week wash-out period; interestingly, during the IGF-1 therapy phase a significant improvement in both social impairment and restrictive behaviors was demonstrated. To the best of our knowledge, no studies have been conducted on recombinant human Growth Hormone (rhGH) therapy in AT/RT and PMDS and/or r22, this is the first case with GHD treated with hrGH described in the literature.

In the reported clinical case, hrGH therapy showed improvement in the growth rate despite an initial low dosage chosen for safety purposes, body thinness and spinal irradiation. It is important to underline that hrGH therapy improved the quality of life, as reported by the parents and demonstrated by a better resistance to rehabilitation stresses, recovery of dysphagia for fluids and increased muscle mass.

In consideration of the multiple procedural anesthesia, essential for radiological follow-up, the involvement of the multidisciplinary team, turned out to be crucial for reducing the risks associated with sedation. Indeed, *Shank3* mutation confers enhanced sensitivity to isoflurane volatile anesthetic in central nervous systems of mutant mice models (16). This aspect deserves further study considering the important clinical implications, such as to avoid excessive use of volatile anesthetics, and suggests that this vulnerable set of patients may need additional monitoring during anesthesia.

## Conclusions

At the end of this report there are some issues worth further consideration. To our knowledge, this is the first reported patient with PMDS and r22 that survived a childhood metastatic AT/RT. Furthermore, he is the third reported case of r22 and 22q13 deletion not including *SMARCB1* associated to this rare malignant tumor (8, 11). This underlines the importance of regular screening for early detection of central nervous system rhabdoid tumors in patients with r22 and deletion of 22q13 (although not involving mutations of the *SMARCB1* gene). The hrGH therapy was safe, it improved the auxological parameters and the quality of life of the entire family; however, GHD must be ruled out early when the growth velocity declines, also in the context of a normal height, in order to initiate hrGH therapy as soon as the oncological conditions allow it. The management of GHD in high-grade rhabdoid tumors is still under debate. In accordance with international guidelines, especially in the context of genetic cancer predisposition syndromes, a multidisciplinary discussion is mandatory. The GHD in cancer survivors could be treated with the same hrGH doses as is used for non-cancer GHD patients.

Finally, we would like to suggest the importance of studying and defining a protocol to reduce the potential risks related to the administration of volatile anesthetics in PMDS.

Following the analysis of Byers et al. (11), it could be argued that ring chromosome 22 may be decisive for the occurrence of AT/RT when *SMARCB1* is not constitutionally mutated, however, further histogenetic analyses are necessary to explore this hypothesis, and the possible involvement of other contiguous genes.

## Data Availability Statement

The original contributions presented in the study are included in the article/supplementary material, further inquiries can be directed to the corresponding author/s.

## Ethics Statement

Ethical review and approval was not required for the study on human participants in accordance with the local legislation and institutional requirements. Written informed consent to participate in this study was provided by the participants' legal guardian/next of kin. Written informed consent was obtained from the individual(s), and minor(s)' legal guardian/next of kin, for the publication of any potentially identifiable images or data included in this article.

## Author Contributions

MC and MP designed the study, collected the data, drafted and revised the manuscript. ND and MG took care of the patients and revised the manuscript. AV, CMi, and CMo helped in the evaluation and the following-up of the patient. VC actively participated in the data analysis, drafting, and revision of the manuscript. All authors approved the final manuscript as submitted and agree to be accountable for all aspects of the work.

## Conflict of Interest

The authors declare that the research was conducted in the absence of any commercial or financial relationships that could be construed as a potential conflict of interest.

## Publisher's Note

All claims expressed in this article are solely those of the authors and do not necessarily represent those of their affiliated organizations, or those of the publisher, the editors and the reviewers. Any product that may be evaluated in this article, or claim that may be made by its manufacturer, is not guaranteed or endorsed by the publisher.
